# E3 ubiquitin ligase on the biological properties of hematopoietic stem cell

**DOI:** 10.1007/s00109-023-02315-6

**Published:** 2023-04-21

**Authors:** Qianru Zhan, Jing Wang, Heyang Zhang, Lijun Zhang

**Affiliations:** Department of Hematology, The First Hospital of China Medical University, No. 155, Nanjing North Street, Shenyang, Liaoning People’s Republic of China

**Keywords:** Hematopoietic stem cell, E3 ubiquitin ligases, Quiescence, Self-renewal, Lineage differentiation, Homing ability

## Abstract

Hematopoietic stem cells are a group of heterogeneity cells with the potential to differentiate into various types of mature blood cells. Their basic biological properties include quiescence, self-renewal, multilineage differentiation, and homing ability, with the homing of exogenous hematopoietic stem cells after transplantation becoming a new focus, while the first three properties share some similarity in mechanism due to connectivity. In various complex mechanisms, the role of E3 ubiquitin ligases in hematopoietic homeostasis and malignant transformation is receiving increasing attention. As a unique part, E3 ubiquitin ligases play an important role in physiological regulation mechanism of posttranslational modification. In this review, we focus on the recent progress of the crucial role of E3 ubiquitin ligases that target specific proteins for ubiquitination to regulate biological properties of hematopoietic stem cells. Additionally, this paper deals with E3 ubiquitin ligases that affect the biological properties through aging and summarizes the relevant applications of targeting E3 ligases in hematopoietic malignancies. We present some ideas on the clinical application of E3 ubiquitin ligase to regulate hematopoietic stem cells and also believe that it is meaningful to study the upstream signal of these E3 ubiquitin ligases because hematopoietic stem cell dysfunction is caused by deficiency of some E3 ligases.

## Introduction

**Fig. 1:**
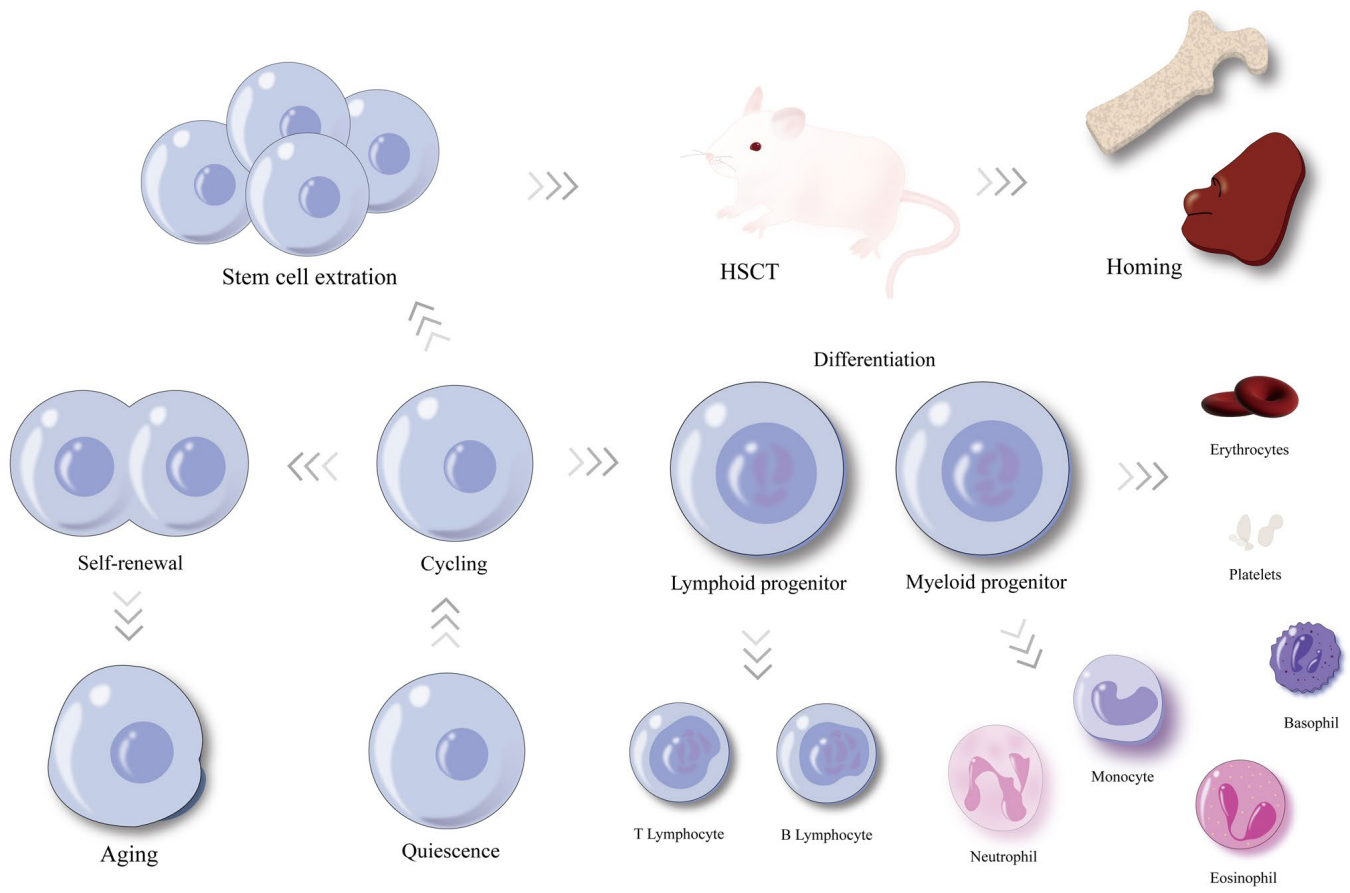
The process of hematopoiesis: an intricate balance among quiescence, cycling, self-renewal, lineage differentiation, aging, and homing

Hematopoietic stem cells (HSCs) are the cradle of mature blood cells. Their basic biological properties include quiescence maintenance, self-renewal, multilineage differentiation, and homing ability (Fig. 1). The capability of self-renewal can maintain the permanent repopulation of most hemopoietic cell lineages, and lineage differentiation means that HSCs can differentiate into progenitor cells under certain conditions. In most cases, HSCs exist in a quiescent state to maintain the function of stem cell compartment and prevent exhaustion, of which the LT-HSCs (long-term HSCs) are more obviously at quiescent state and have more powerful self-renewal capability [1, 2]. HSC homing ability refers to their function of hematopoietic reconstitution in the bone marrow (BM) niche. A series of intrinsic and extrinsic factors can affect hematopoietic biological properties in different ways, involving HSC environment (also named the HSC niche) [3, 4], transcription factors [5–7], cell cycle regulators [8], metabolic pathways [9], and cytokine signaling [10, 11]. In addition, the role of posttranslational modification has gradually come into researchers’ perspective.

For approximately two decades, there has been an increasing number of findings focusing on the effects of posttranslational modification on the hematopoietic system, among which the ubiquitin-dependent proteasome degradation system attracts considerable interest. Ubiquitin (Ub), which is a 76-residue small protein, can be labeled on the surface of specific protein substrates with the aid of enzymes, and then, the targeted proteins can be recognized and degraded by organelles or multienzymatic complexes [12]. There are three main groups of enzymes involved in the Ub system, namely, E1 Ub-activating enzyme, E2 Ub-conjugating enzyme, and E3 Ub-ligating enzyme. It is E3 ubiquitin ligases that perform an irreplaceable role in recognizing and binding specific target proteins. Aberrant ubiquitination or disruption of the balance between ubiquitination and deubiquitination can transform the physiological condition into a pathological state, especially causing cancer [13]. It is precisely because of their high substrate specificity that E3 ubiquitin ligases are promising therapeutic targets for cancer treatment. Currently, anticancer drugs targeting the E3 ligases have been actively developed, and their therapeutic effects have been suggested by animal experiments and clinical trials [14, 15].

**Fig. 2:**
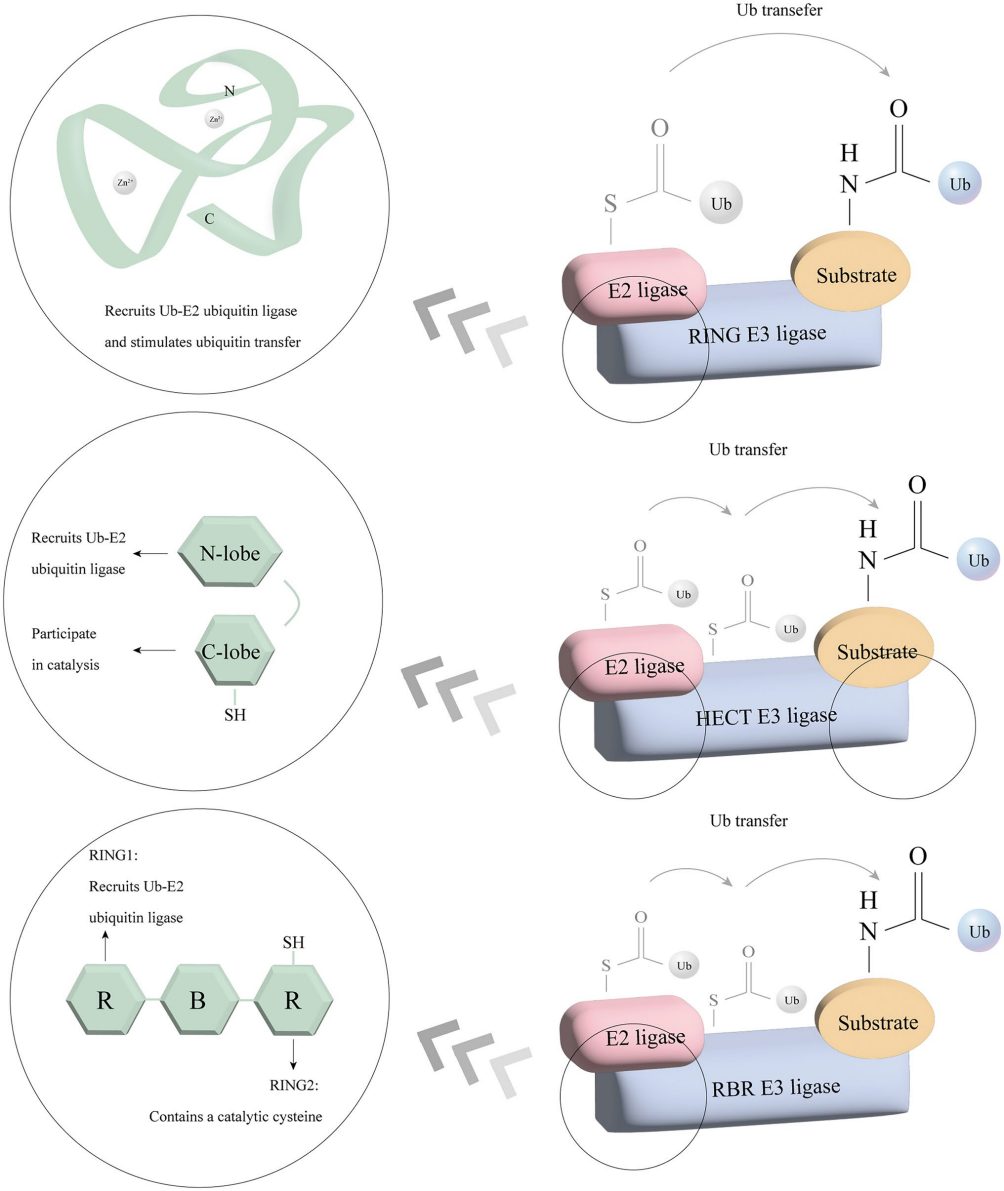
The classification of E3 ubiquitin ligases

There are more than 600 types of E3 ubiquitin ligases that have been found. Classically, these E3 can be classified into three main types, RING (really interesting new gene) type, HECT (homologous to E6AP C-terminus) type, and RBR (RING-between-RING) type, respectively [16, 17] (Fig. 2). Two major ubiquitin transfer mechanisms were revealed among these three E3 types, based on their different structures and biochemical properties. The direct transfer from the complex of E2 and ubiquitin to the substrate can be catalyzed by RING type E3, while HECT and RBR type E3 have a receive-and-transfer process from the complex of E2 and ubiquitin to the substrate [18]. As the smallest, RING domain of RING E3s harbors two zinc ions, providing the Zn coordination in a cross-braced configuration for domain folding [19]. RING E3s exerted their E3 activity with a highly diverse quaternary architecture and five principal biological forms of assembly: monomer (such as CBL and RNF38), homodimer (such as TRAF6 and RNF4), heterodimer (such as TRIM family), oligomer, and component of multi-subunits (such as CRL and APC/C) [20–25]. Besides, the U-box domain is the same as the RING fold, but without zinc. For the HECT E3s, they consist of an N-terminal substrate-binding domain and a C-terminal HECT domain. The C-terminal HECT domain was first discovered in human papillomavirus E6-associated protein (E6AP) 5, containing almost 350 amino acids [26, 27]; there are two lobes in the conserved HECT domain that are connected by a flexible hinge loop, with the N-terminal lobe (N-lobe) binding to E2 ~ ubiquitin and the C-terminal lobe (C-lobe) having the catalytic cysteine residue [28]. Based on the different N-terminal domains, HECT E3s can be divided into NEDD4 family, HERC family, and HECTs with other protein–protein interaction domains [29].

Here, we specifically summarize the E3 ligases which affect the biological properties of HSCs, analyzing those containing Cbl (Casitas B-cell lymphoma), Fbxw7 (F-box and WD-40 repeat domain-containing protein 7), Skp2 (S-phase kinase-associated protein 2), Maea (macrophage erythroblast attacher), Itch, Triad1 (two RING fingers and DRIL 1), Ddb1 (damage-specific DNA-binding protein 1), ASB2α (Ankyrin Repeat and SOCS Box-Containing 2α), Smurf2 (SMAD-specific E3 ubiquitin protein ligase 2), Mdm2 (murine double minute2), and VHL (von Hippel-Lindau protein) (Table 1). Besides, the E3 ubiquitin ligase Mdm2, which indirectly affects the biological characteristics by influencing the aging of HSCs, is involved in this review, and the related utilization of targeting E3 ubiquitin ligases in hematopoietic malignancy is also investigated. It is worth noting that hematopoietic stem cell transplantation (HSCT) can obviously improve the overall survival and disease-free survival in the majority of malignant hematologic diseases compared with traditional chemotherapy. The regimen of HSCT combined with chemotherapy has received wide attention, used to cure or ameliorate a number of hematologic and genetic disorders. Therefore, understanding the molecular mechanisms that control hematopoietic biological properties is beneficial for targeted therapy development and then improve the effectiveness of therapeutic approaches.

**Table 1:** E3 ligases affect the function of hematopoietic stem cells

E3 ligases	Affected functions	Related mechanism
Asb2α^108^	Binding/homing	• HSC adhesion (binding ability) in an integrin-dependent manner in the absence of FLNa
Skp2^47,109^	Binding/homing	• HSC adhesion (binding ability) in a collagen I and fibronectin-dependent manner • β-Catenin is a downstream executor of Skp2 affecting the HSC homing ability
	Quiescence	• Targeting p27 and p21 for ubiquitination and degradation, then trigger cell cycle progression • Cyclin D1 knockdown remarkably reduced the G1 population of Skp2-deficient LSK cells
Maea^48^	Quiescence	• mTOR activity suppressed by Maea guarding HSC quiescence
Cbl^43,82^	Quiescence	• The expression level of p57 and p21 was significantly reduced in Cbl/Cbl-b double-knockout mice, which are essential for maintenance of HSC quiescence • Impaired ligand-induced c-Kit and FLT3 downregulation • Hyperactive PI3K signaling pathway (showing as sustained AKT and S6 activation)
	Differentiation	• Function as a negative regulator in HSC development • STAT5 phosphorylation was increased and c-Myc transcripts were enhanced in c-Cbl-deficient HSCs, indicating c-Cbl-mediated ubiquitination is critical for TPO signaling in HSCs
Fbxw7^56,77–78^	Quiescence	• c-Myc and Notch1 proteins were increased in Fbxw7-deficient mouse LSK cells, which are related to cell cycle progression
	Self-renewal	–
	Differentiation	• GATA2 levels were increased in HSCs and myeloid progenitors in conditional Fbxw7-deficient mice, with a decreased c-Kit high expressing population of myeloid progenitor cells • Affecting T cell development (T cell progenitors are unable to colonize the thymus under Fbxw7 deficiency)
Itch^69^	Self-renewal	• The Notch1 protein level is higher in Itch-deficient LT-HSCs with more cleaved Notch1
Triad1^84^	Differentiation	• The inhibition role of Triad1 in the development of immature progenitors toward mature myeloid cells, probably caused by a cell cycle arrest
FLRF^88^	Differentiation	• Elevated FLRF protein level attenuates EML cells differentiated into myeloid and erythroid lineages by downregulating the EPO, IL-3, and RA receptors
Ddb1^85^	Differentiation	–
Smurf2^129−130^	Self-renewal	–
	Senescence/aging	–
Mdm2^127^	Senescence/aging	• Mdm2 can target and degrade p53 to keep p53 at a low level • Mdm2-deficient p53^515C/515C^ mice showed an increased aging cell

## E3 ubiquitin ligases and their effects on the biological properties of HSCs

### E3 ubiquitin ligases and their effects on quiescence of HSCs

Cell cycle progression can be divided into four phases: gap 1 (G_1_), DNA synthesis (S), gap 2 (G_2_), and mitosis (M) [30]. Quiescence is a state of reversible cell cycle arrest, in which cells enter G_0_ instead of participating in the cell cycle. Under the proper stimuli, quiescent cells can make a response and reenter the cycling state [31]. Quiescence has long been viewed as a dormant, low-activity state, but now it is more likely to be believed that quiescent cells are poised with potential and active restraint [32, 33]. A large pool of HSCs in a quiescent state can assure long-term hematopoietic capacity. Loss of quiescence can cause HSC exhaustion, rendering long-term reconstitution impossible [34]. Some critical growth factors are necessary for maintenance of quiescence among HSCs, such as stem cell factor (SCF), angiopoietin-1, and thrombopoietin (TPO), and the associated receptors are c-Kit, Tie-2, and c-Mpl, respectively [35–38]. The leukemogenesis may occur when the receptors or their downstream protein tyrosine kinases (PTKs) have mutational activation [39]. Additionally, the Cip/Kip family of cyclin-dependent kinase (CDK) inhibitors, including p57, p27, and p21, functions as cell cycle regulators to maintain HSC quiescence [40, 41]. Furthermore, LT-HSCs can remain in quiescence through interacting with the BM niche [42] p57 is predominantly expressed in the LT-HSCs, which is essential for maintenance of HSC quiescence (rather than p27 and p21) [40]. According to Wei et al. [43], the levels of expression of p57 and p21 were significantly reduced in Cbl/Cbl-b double knockout (DKO) mice LT-HSCs compared to wild-type (WT) HSCs, suggesting reduced quiescent LT-HSCs in DKO mice. Based on cell cycle status, DKO LSKs (lineage^−^Sca-1^+^c-Kit^+^) showed a significant decrease in G_0_ phase, while DKO LSKs in the G_2_ and G_2_/S phases were enhanced [43]. Besides, impaired ligand-induced c-Kit and FLT3 downregulation (only c-Kit downregulation was statistically significant) and sustained downstream signaling in DKO LSKs proved that Cbl proteins negatively regulate receptor tyrosine kinases (RTKs) in normal LSKs [43]. DKO LSKs present hyperproliferation in response to SCF and FLT3L [43]. Furthermore, the hyperactive PI3K signaling pathway (showing as sustained AKT and S6 activation) may be one cause of reduced quiescence in DKO LSKs [43]. Skp2 is a member of the class of F-box proteins. The Skp2 SCF complex, which is composed of Skp1, Cullin-1, and Rbx1, can target p27 and p21 for ubiquitination and degradation and then trigger cell cycle progression [44–46]. As per Wang et al. [47], the numbers of Skp2-deficient LSK cells and GMP populations are higher than those of matched WT LSK cells, indicating that Skp2 is a negative regulator. Further studies displayed an increase of Skp2-deficient LT-HSCs in the G_2_/M phase and a decrease in G_1_/G_0_ phase, indicating Skp2 deficiency promotes LT-HSC cycling and proliferation, which may be the cause of increased HSC pool. Taken together, Skp2 maintains HSCs in the quiescent stage, preventing them from entering the cycling phase. The potential mechanism may be related to Cyclin D1, as knockdown of this gene remarkably reduced the G_1_ population of Skp2-deficient LSK cells. Wei et al. [48] constructed Maea deletion mice using the conditional Mx1-Cre line (Maea^Mx1−Cre^) and poly I:C administration. In these Maea-deficient mice, the quiescent HSCs were lost to a great extent but this was not the case among other cell populations. After 4 months, mild anemia, myeloid expansion, and significantly decreased BM HSC numbers were detected. Moreover, the cycling HSCs were more active in young Maea^Mx1−Cre^ mice than in older mice. Both results suggest that Maea deletion depletes HSCs. Further study confirmed that mTOR activity is suppressed by Maea guarding HSC quiescence [48]. Fbxw7 can target a series of substrates for ubiquitination and degradation, including Notch1, c-Myc, and GATA2, which are related to cell cycle progression [49–53]; Myc also has an essential role in cell growth and self-renewal [54, 55]. In Fbxw7-deficient LSK cells, c-Myc and Notch1 proteins were increased [56]. In this report, the author proved that the numbers of leukocytes, hemoglobin, platelets, and LSK cells were decreased in Fbxw7-deficient mice, and depletion of normal BM HSCs was caused by cell cycle promotion. In another study, the percentage of LSK cells entering cell cycle was significantly elevated. Consistently, Fbxw7-deficient LT-HSCs showed a dramatic loss of quiescence with 80% of them entering the cell cycle [57]. Besides gene deletion, heterozygous Fbxw7 mutations can affect the frequency and absolute number of HSCs, with significant lower numbers in Fbxw7^F/+^Mx1-Cre + (Fbxw7^Δ/+^) bone marrow compared to Fbxw7^mut/+^Mx1-Cre + and Fbxw7^F/F^Mx1-Cre + (Fbxw7^Δ/Δ^) mice [58]. In normal BM niche, HSCs present with intracellular hypoxia and stabilized hypoxia-inducible factor-1α (HIF-1α), a transcriptional regulator that can maintain HSCs at the quiescence level [59]. However, biallelic loss of the E3 ubiquitin ligase VHL can lead to HIF-1α overstabilization, inducing cell cycle quiescence in HSCs and progenitors but transplantation capacity impairment [59]. In contrast, monoallelic loss of VHL can induce quiescence and improve BM transplantation [59].

**Fig. 3:**
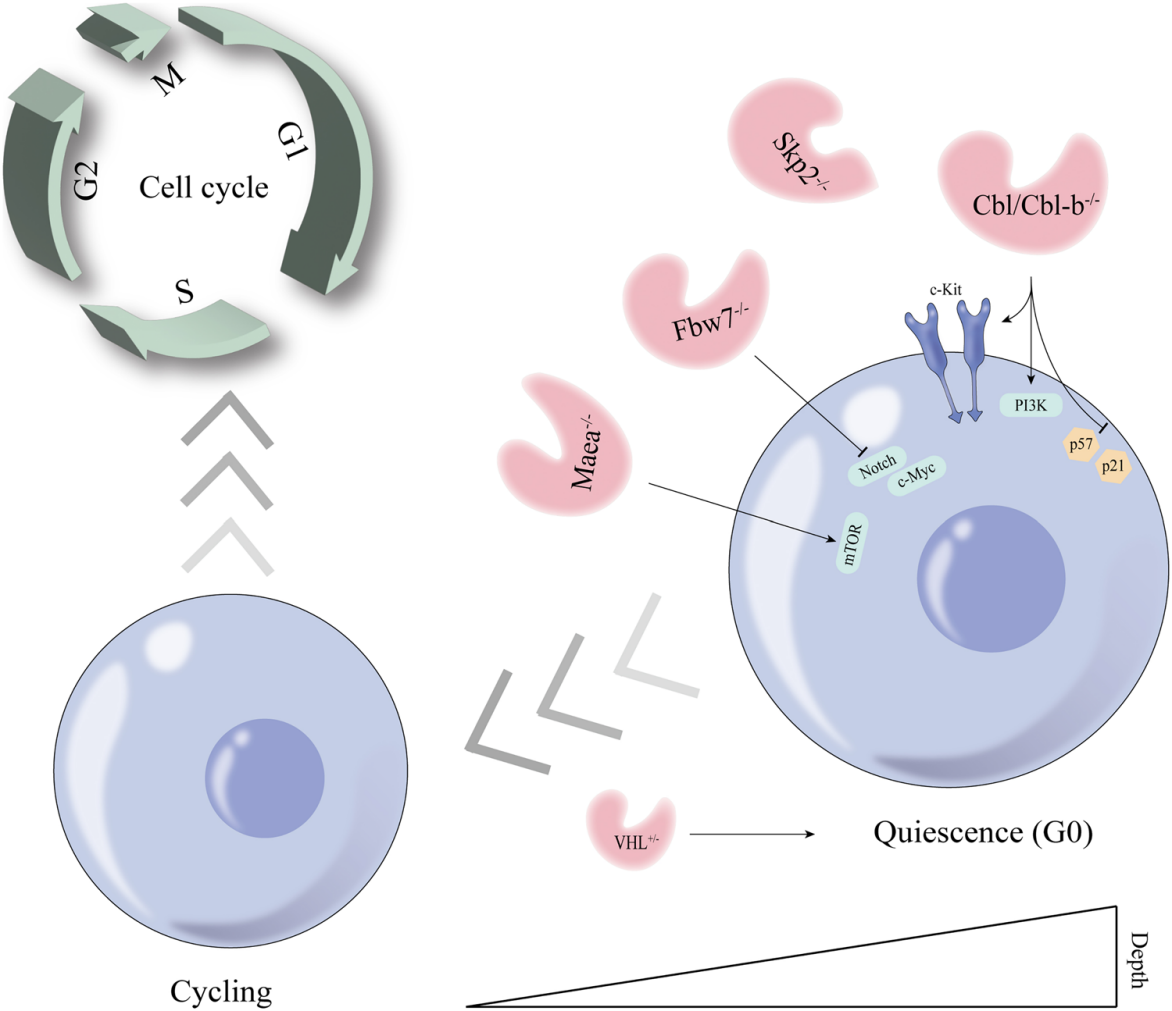
The regulation between E3 ubiquitin ligases and hematopoietic stem cell quiescent state. Deficiency of E3 ligases (Cbl/Cbl-b, Skp2, Maea, and Fbxw7) disrupts the quiescent state of hematopoietic stem cells, increasing the cycling state. Monoallelic loss of VHL increases the state of quiescence

In conclusion, given that the quiescence state is the basis for self-renewal and multilineage differentiation, the effect of E3 ubiquitin ligase on this state is likely to cause alterations in cell cycle (Fig. 3), thus triggering different degrees of alterations in the ability to self-renew and differentiate. Therefore, the regulation of the quiescence state is particularly important. For the clinical application of HSCT, such regulation may provide a new idea about HSC mobilization before patients receive HSCT. In malignant hematological diseases such as leukemia, the blasts have been transformed from HSCs to leukemic stem cells, and it will provide a new targeting idea if the leukemic stem cells are overactivated or overquiescent.

### E3 ubiquitin ligases and their effect on self-renewal of HSCs

The self-renewal property of HSCs was maintained through asymmetrical mitosis. When HSCs out of quiescence state and enter the cell cycle, the asymmetrical mitosis would response in order to achieve self-renewal. It is strictly regulated by intracellular and extracellular factors, such as the bone marrow niche, cytokines, transcription factors, signal transducers, cell cycle inhibitors, and surface receptors [60–66]. For instance, significant expansion of multipotential progenitors and promotion of HSC self-renewal in vitro can be detected in STAT5-activated LT-HSCs [67]. It is worth noting that because of the continuity between self-renewal and the quiescence maintenance, the mechanisms of both are strikingly similar in some respects.

The HECT-type E3 Itch ligase plays a critical role in hematopoietic development. According to Rathinam et al. [68], Itch-deficient bone marrow cells can protect recipients from lethal irradiation, even in the later stages of life (20 weeks). As for the long-term repopulation abilities, serial competitive transplantation presented with greater ability in Itch-deficient mice. Moreover, the recovery capability can be enhanced under stress conditions. It is confirmed that Itch is involved in Notch1 ubiquitination, and there are numerous similarities in phenotypic characterization between Itch-deficient HSCs and Notch1-overexpressing HSCs [68, 69]. Notch1 is a transmembrane protein that serves as a ligand-activated transcription factor. The expression of Notch1 can be detected in BM precursor cells, peripheral T and B lymphocytes, monocytes, and neutrophils [70]. Research shows that constitutive Notch1 signaling regulates HSC self-renewal, immortalizing in vitro cell lines, although it may need a second mutation [71]. Besides, activated Notch1 signaling inhibits HSC differentiation (in vitro and in vivo), enhancing self-renewal among stem cells [70]. The mechanism research demonstrated that the Notch1 protein level is higher in Itch-deficient LT-HSCs in comparison to WT LT-HSCs and more cleaved Notch1 in Itch-deficient LT-HSCs as well (both in the nucleus and cytoplasm) [68]. In line with this, the Notch signal is augmented in Itch-deficient HSCs isolated from Itch-deficient transgenic Notch reporter mice; however, the Notch1 mRNA level was not significantly different in Itch-deficient LT-HSCs and WT LT-HSCs [68]. The inconsistent level of mRNA and protein indicates that the increased Notch1 protein is the consequence of defective degradation. Further research showed that Notch1 protein was ubiquitinated only in the presence of Itch; the knockdown of Notch1 decreased the frequency of HSCs and diminished the radioprotective function thereof [68]. According to the results of the quiescence part, in Fbxw7-deficient mice, the LSK quiescence was decreased, while most LT-HSCs entered the cell cycle [57], and the expression level of c-Myc protein increased [56]. Meanwhile, the reason in part for this self-renewal deficiency in Fbxw7-deficient HSCs is an aberrant accumulation of c-Myc protein, while the level of c-Myc stabilization in Fbxw7-mutant HSCs is slightly lower than that in Fbxw7-deficient HSCs; this intermediate level has no functional effect on self-renewal [58, 72].

**Fig. 4:**
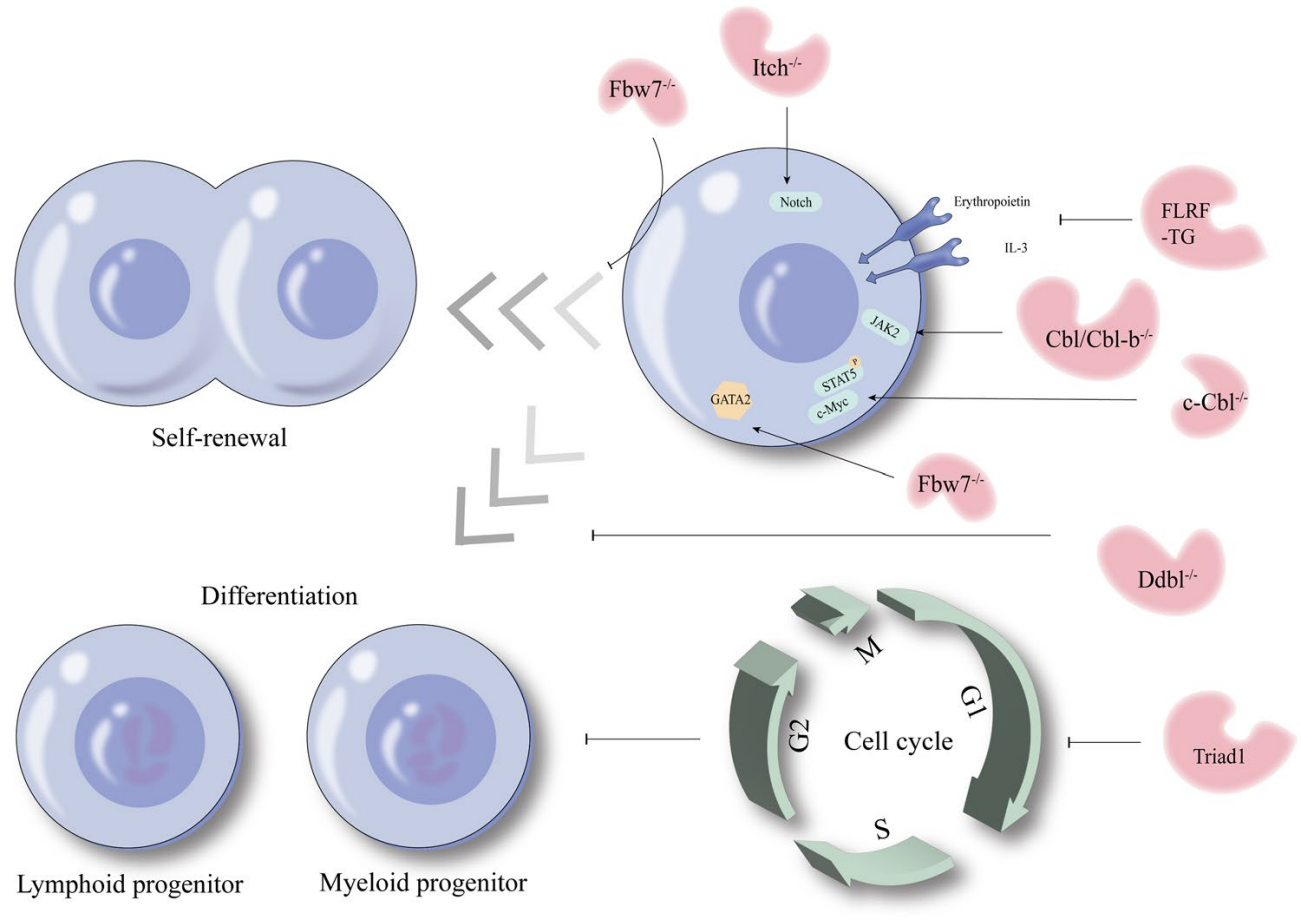
The regulation between E3 ubiquitin ligases and hematopoietic stem cell self-renewal or hematopoietic stem cell differentiation. Deficiency of Fbxw7 disrupts the self-renewal of hematopoietic stem cell, while deficiency of Itch can enhance this ability. As to the hematopoietic stem cell differentiation, deficiency of Fbxw7 can inhibit T lymphoid progenitor; deficiency of Cbl/Cbl-b can enhance myeloid expansion, while Triad1 plays an inhibitory role in the development of immature progenitors toward mature myeloid cells. In addition, overexpression of FLRF in EML cell lines suppresses the myeloid and erythroid lineage differentiation

In general, Notch1 signaling seems to be important in the Itch-participated regulation of self-renewal capability. Therefore, there are one-sided assumptions. The development of the short-acting Itch-Notch1 targeted drug can be research and development; it will contribute to long-term hematopoiesis posttransplantation. Besides, the targeted agonists of Fbxw7 may enhance the self-renewal capability of HSCs. However, it is worth pondering that the HSCs entering the cell cycle in Fbxw7-deficient mice do not enhance their self-renewal ability as a result; instead, this uncontrolled cell cycle state may result in dysfunctional self-renewal (Fig. 4).

### Effect of E3 ubiquitin ligases on lineage differentiation of HSCs

Lineage differentiation of HSC, multipotential progenitors, and committed progenitors was regulated by diverse factors, including cytokine receptor levels, cell cycle arrest, and noncoding RNAs [73–76]. What is clear is that the mechanisms affecting lineage differentiation are also strongly linked to the quiescence state and self-renewal as well. For example, although it is inhibitory on HSC differentiation, activated Notch1 signaling promotes common lymphoid progenitors, early natural killer (NK), and T-progenitor cell differentiation, at the expense of myeloid development [68].

The transcription factor GATA2 participates in proliferation and differentiation of HSCs, and its expression level is affected by cell cycle, with high levels in the S phase but low in G1/S and M phases [52]. The levels of GATA2 were increased in HSCs and myeloid progenitors in conditional Fbxw7-deficient mice, this condition being correlated with a decreased c-Kit expression among the population of myeloid progenitor cells [77]. As to the development of T cells, Fbxw7 expression is higher in T cell-committed progenitors than in B cell progenitors [57]. Furthermore, T cell progenitors are unable to colonize the thymus under Fbxw7 deficiency, causing depletion of T cell progenitors, and CD8 single-positive lineage was skewed under Fbxw7 conditional inactivation [57, 78]. The proto-oncogene c-Myc is important not only for self-renewal, but also for differentiation [79, 80]. As an E3 ligase, c-Cbl mRNA level was detected in all types of HSC subsets with maximal expression in the LT-HSCs [81]. Meanwhile, the numbers of LSK fraction and LT-HSCs were markedly increased in c-Cbl-deficient mice. Through measuring the number of different subsets, it can be found that compared with WT mice, c-Cbl-deficient mice had increased absolute numbers of LT-HSCs, ST-HSCs, and multipotent hematopoietic progenitor cells (MPP), but no significant difference in other differentiated hematopoietic lineages. It appears that MPP is more affected. At the molecular level, STAT5 phosphorylation was increased and c-Myc transcripts were enhanced in c-Cbl-deficient HSCs, indicating c-Cbl-mediated ubiquitination is critical for TPO signaling in HSCs [81]. In addition, research proved that Cbl/Cbl-b or LNK (also called SH2B3) ex vivo deletion extends JAK2 half-life through the CBL-LNK-JAK2 signaling axis, enhancing HSC reconstitution and myeloid expansion [82]. JAK2 is known as an essential role in hematopoietic development, and uncontrolled JAK2 signaling results in hematopoietic malignancies [83]. Fresh bone marrow and leukapheresis samples were stained to detect the expression of Triad1, discovering Triad1 is expressed only at low levels in immature progenitors (CD34^+^) and T cells (CD3^+^) while being highly expressed in granulocytes (CD15^+^) and monocytes (CD14^+^) [84]. The morphological analysis of clonogenic growth in semisolid medium discovered that the percentage of granulocytes and the percentage of erythroblasts slightly decreased. Additionally, in cellular experiment, significant inhibition of cell proliferation and an increased cell apoptosis can be detected in Triad1 retrovirally transduced BM cells, with a marked increase in the G_0_/G_1_ phase. These observations suggested an inhibitory role for Triad1 in the development of immature progenitors toward mature myeloid cells, probably caused by a cell cycle arrest. DNA damage-binding protein 1 (Ddb1) belongs to a component of the Cullin4-containing E3 ubiquitin ligase. As Ddb1-deficient BM cells cannot form colonies in cytokine-supplemented culture as well as in spleen of host mice, it was revealed that the differentiation ability of Ddb1-deficient HSCs was impaired [85].

Many studies indicate that the hematopoietic cell differentiation is affected by the predetermined amount of related receptor expression, partially explained by the signaling-threshold model [86, 87]. Before the receptor binds to the ligand, the optimal number of receptor levels ensures cellular proliferation and differentiation. Jing et al. [88] experimentally investigated E3 ligase FLRF using erythroid-myeloid-lymphoid (EML) cell line, which is a multipotent hematopoietic cell line with a latent ability of multilineage differentiation. In hematopoietic colony-forming assays, compared with WT EML and vector control EML/pcDNA cells, the colonies (CFU-GM, BFU-E, and CFU-Meg) of EML/FLRF cells generated three to fivefold lower numbers, indicating FLRF overexpression attenuates myeloerythroid differentiation. Erythropoietin induces BM-derived hematopoietic progenitors and EML cell line differentiated into erythrocyte lineage. Interleukin 3 (IL-3)-induced differentiation of BM-derived hematopoietic progenitors and EML cell line into granulocyte and macrophage lineages and retinoic acid (RA) can amplify this effect [89]. There are also studies demonstrating that IL-3 and granulocyte–macrophage colony-stimulating factor signaling can activate RA receptor RARα through the JAK2/STAT5 pathway [90, 91]. After analyzing the levels of erythropoietin and IL-3 receptors, they found that they were significantly decreased in EML/FLRF cells both before and after exposure to erythropoietin and IL-3. Since ligand stimulation leads to receptor internalization and degradation, FLRF may play an important role in ligand-independent receptor degradation. Interestingly, treatment with RA further impaired IL-3-induced differentiation into CFU-GM progenitors. Immunoprecipitation and western blotting found that the protein level of RA receptor RARα was significantly reduced while the RA receptor retinoid X receptor protein levels remained unchanged. In conclusion, elevated FLRF protein level attenuates EML cells differentiated into myeloid and erythroid lineages by downregulating the erythropoietin, IL-3, and RA receptors.

In summary, compared with the quiescence state and self-renewal ability, lineage differentiation seems to involve more equilibrium (Fig. 4). Ligand-independent receptor degradation is a good idea for drug development. In the future, under the condition of mature technology in this field, the sensitivity of HSCs to hematopoietic stimulating factors can be altered by receptor regulation, thus contributing to hematopoietic regulation and HSCT.

### E3 ubiquitin ligases and their effects on homing of HSCs

The homing ability means that HSCs can home to BM and spleen, which is vital for lifetime hematopoiesis [92, 93]. The significance of homing in the physiological state remains unclear, and more studies have focused on the homing of exogenous hematopoietic stem cells after receiving HSCT. It can be influenced by numerous uncertain factors, including stem cell niche and the binding of HSCs to their niche. The binding ability is regulated by collagen I and fibronectin, which can act as adhesion molecules by providing the endosteal surface of bone [94]. In addition to these, there are many adhesion molecules supposed to be involved in the regulation of HSC homing, such as integrins [95], selectins [96–99], and N-Cadherin [100].

**Fig. 5:**
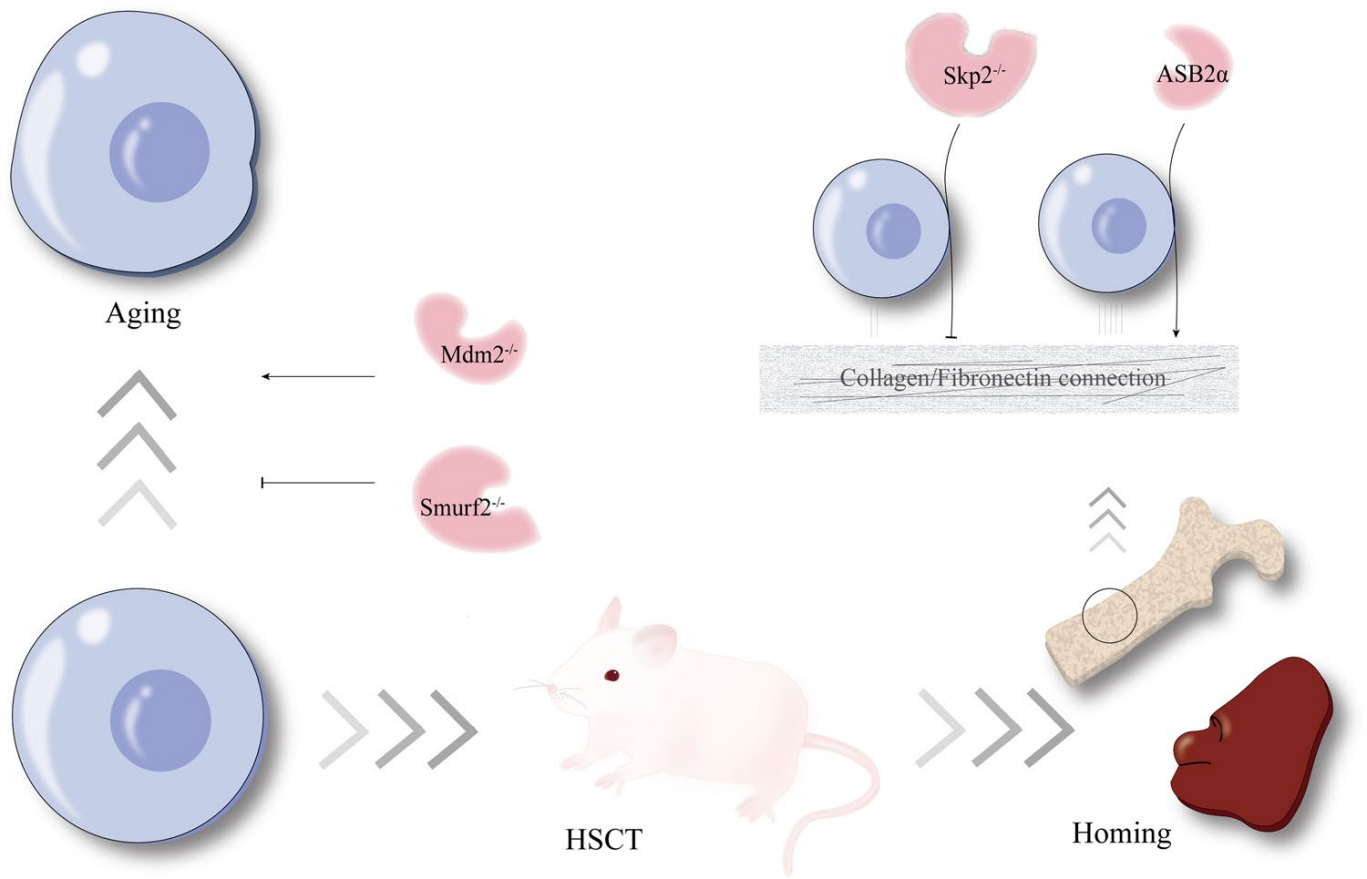
The regulation between E3 ubiquitin ligases and hematopoietic stem cell homing or hematopoietic stem cell aging. The E3 ligases Skp2 and ASB2α can regulate the homing ability through adhesion. On the other hand, deficiency of Mdm2 promotes the aging of hematopoietic stem cells, while a deficiency of Smurf2 can interrupt the process, affecting the biological function in different degrees

Skp2 is a component of the Skp2-SCF E3 ligase complex. Although the HSC pool was enhanced and long-term reconstitution ability was increased in Skp2-deficient mice, study has shown that the homing ability of HSCs to BM and spleen was markedly reduced [46, 101]. This study observed that Skp2-deficient BM cells demonstrated a significant reduction in BM niche and spleen niche in vivo experiments and that Skp2-deficient LSK cells had reduced adhesion to collagen I and fibronectin (mimicking the bone marrow microenvironment) in vitro 96-well plates, suggesting that Skp2 is an essential factor for HSC homing [101]. Through an unbiased microarray analysis, a critical gene β-catenin was downregulated in Skp2-deficient BM cells. Besides, the target genes of β-catenin including MMP7, c-Myc, CD44, NF-kB, and Axin2 were reduced as well [101]. Knockdown of β-catenin in WT mice reduced the WT HSC homing ability as well, which mimicked the decline of HSC homing upon Skp2 deficiency [101]. It was confirmed that β-catenin is a downstream executor of Skp2 affecting the HSC homing ability. However, the question is that the experiment was performed with BM cells for transplantation and LSK cells for adhesion experiments, neither of which were pure HSCs. Is it true that the decrease in homing ability of BM cells is proportional to the decrease in homing ability of HSCs? There could be various influences on this, such as mesenchymal stem cells. In addition, integrin adhesion molecules are essential for HSCs to anchor in the hematopoietic niche [102, 103]. ASB2, a retinoic acid response gene, is identified as a target gene for PML-RARα fusion protein in acute promyelocytic leukemia [104, 105]. Recently, it is confirmed that the hematopoietic-type isoform ASB2α can ubiquitinate and degrade an actin-binding protein filamin A (FLNa), making HSC adhesion in an integrin-dependent manner in the absence of FLNa [106–108]. To be more specific, ASB2α enhances adhesion of hematopoietic cells to fibronectin (Fig. 5), which is the main ligand of β1 integrins.

Overall, there are conflicting views on whether Skp2 deletion results in enhanced long-term reconstitution capacity. According to Rodriguez et al. [109], the long-term reconstitution capacity of HSCs is comparable between WT and Skp2-deficient mice, and only short-term hematopoietic recovery was a bid of defective. Homing ability affects the posttransplant reconstitution to a large extent, but it does not directly determine the reconstitution. The long-term hematopoietic reconstitution in Skp2-deficient mice after transplantation was not statistically different from that in WT mice, possibly because Skp2-deficient HSCs are inherently more capable of self-renewal, more sensitive to hematopoietic stimulating factors in the later stages of hematopoietic reconstitution, or for other reasons. There may exist two independent ways to modulated homing and hematopoietic reconstitution. Furthermore, although transplantation with BM cells does not fully reflect and explain the homing properties of HSCs, the finding of an enlarged pool of HSCs with a diminished homing capacity is of interest.

## E3 ubiquitin ligases and their effects on biological properties of HSCs by regulating aging

The concept of HSC senescence seems to be somewhat contradictory to self-renewal. For senescence, it is argued that with each cell division the potential of HSCs to promote blood cell production decreases, while the pool of stem cells with reduced potential increases to compensate for the loss of function of individual stem cell [110]. Therefore, the potential of each daughter HSC is inferior to that of the predecessor stem cells [110]. Whether the aging of HSCs is more influenced by their own factors or by external disturbances remains to be studied more. At present, the more clearly identified influencing factors are mechanistically interdependent and highly interrelated, including DNA damage [111–113], increased polarity [114, 115], impaired autophagy and mitochondrial activity [116–118], and epigenetic reprogramming [119–121]. Aging HSCs accumulate features of extensive DNA damage, including γ-H2AX foci [122], and the extent to which normal HSC senescence is caused by genetic damage is unclear. The most obvious, or most studied, biological property of HSCs subject to aging is their ability to self-renew. For young mouse recipients receiving serial transplants, stem cells derived from BM of older mice have a much lower self-renewal capacity compared to those of young mice [123]. Throughout all transplantation studies, HSCs from young mice competed with older stem cells, and the younger stem cells were invariably functionally superior [124–126].

As an E3 ubiquitin ligase, Mdm2 can target and degrade p53 to keep p53 at a low level. In Mdm2-deficient p53^515C/515C^ mice, β-galactosidase was approximately 11% of cells compared to fewer than 5% of the matched group [127]. β-Galactosidase is a cellular senescence-associated factor [128]. In addition, less than 6.5% Mdm2-deficient p53^515C/515C^ BM cells were in the S phase compared to 22% of the control group (Fig. 5). Research showed that smurf2 plays an important role in the senescence response [129] (Fig. 5). In 24-month C57BL/6 mice, the smurf2 expression was increased compared with 2-month mice [130]. Ramkumar et al. [130] developed a smurf2-deficient mouse model, noticing a statistically significant increase in the number of total live BM cells and the total number of LT-HSCs in 2-month smurf2-deficient mice compared with age-matched WT mice. In 24- to 30-month smurf2-deficient mice, BM cellularity was statistically increased. These results indicate an enhanced cell proliferation of BM cells and LT-HSCs in smurf2-deficient mice, and the research suggested that increased numbers of BM cells arise in the S phase of the cell cycle in 2-month smurf2-deficient mice.

In short, aging is still a point well worth studying. If aging of HSCs by the regulation of E3 ligases can be applied to leukemia stem cells, it would be a significant breakthrough in the treatment of malignant hematologic diseases.

## The utilization of the molecular mechanism of E3 ubiquitin ligase

Hematological disease treatment has progressed from chemotherapy alone to the combination of chemotherapy, targeted therapy, immunotherapy, and HSCT, which represents a significant improvement in prognosis. Among various targets, targeting E3 ubiquitin ligases may be an effective strategy for use as a clinical regimen. Based on the different mechanisms, targeted drugs are mainly divided into four categories: targeted inhibitors of E3 ligases, targeted agonists of E3 ligases, proteolysis targeting chimeras (PROTACs), and molecular glues [131].

Among the E3 ligases that affect hematopoietic malignancies, the most studied inhibitors contain Mdm2, pVHL, Skp2, Itch, and so on, exerting antitumor effects through different mechanisms. Lv et al. [82] found that the regulation of JAK2 by E3 ligase CBL is essential for HSPC lineage development. Ruxolitinib can decrease the number of myeloid-biased and MegE-biased multipotent blood progenitors, indicating that JAK inhibitors can reduce aberrant HSPCs and mitigate leukemia development in Cbl-deficient and Cbl-b-deficient mice [82]. In recent years, PROTACs have attracted much attention, which can remove unwanted or damaged proteins by forming a stable target protein/PROTAC/E3 ternary complex [132, 133]. Besides, the combination of PROTACs and E3 ligases can stabilize tumor proteins and enhance antitumor activity, highlighting the enormous potential in clinical applications [134]. In addition, some well-known drugs, such as cyclosporine A, tacrolimus, and thalidomide, are classical molecular glue examples. This technique can induce a new interaction between E3 ligases and a target protein, which can degrade ligand-free proteins [135, 136].

As for the latest developments in E3 ligases and HSCs, it has been reported that overexpression of Fbxw7 or modulation of the Cullin3-E3 ubiquitin ligase can maintain HSCs ex vivo [137, 138]. Fbxw7-deficient LT-HSCs showed a significant loss of quiescence, while the capability of quiescence is essential for the stemness of HSCs [57]. Hypoxia upregulated the expression of Fbxw7 protein, especially in the nucleus (the isoform of Fbxw7 is preferentially expressed in the nucleus). Overexpression of Fbxw7α presented with high reconstitution capacities in vitro, indicating the importance of Fbxw7α for HSC quiescence [137]. Overall, the modulation of E3 ubiquitin ligases, the exploration of specific E3 ligases and ligands, or the discovery of new molecular glue is promising.

## Discussion

In conclusion, the absence of most E3 ubiquitin ligases may not result in significant incidence of malignant hematological disease but can weaken the HSC ability (quiescence, self-renewal, and homing) or change the tendency for lineage differentiation. So, it is particularly important to combine existing technologies targeting E3 ligases (agonists, inhibitors, PROTAC, and molecular glues) to regulate the biological properties of HSCs. In addition, it is meaningful to study the upstream of these E3 ubiquitin ligases. As HSCT evolves as an irreplaceable treatment, some situations face urgent challenges, involving toxic GVHD, immunological reconstitution after transplantation, the reduction of risk of relapse, and so on. A good knowledge of impaired HSC quiescence, improved HSC self-renewal, and the homing ability could help when taking precautions in advance and then improve the efficacy of stem cell transplantation in the aspect of HSC mobilization, long-term hematopoiesis posttransplantation, and the improvement of the efficiency of HSCT, respectively. E3 ubiquitin ligases could be a predictive marker for monitoring the transplantation efficiency as well. Furthermore, impaired HSC quiescence (overactivated or overquiescent) provides a novel therapy targeting leukemogenic HSCs, and the aging induction can apply to leukemic stem cells as well. There would be a significant breakthrough in the treatment of malignant hematologic diseases, especially acute leukemia.

## Data Availability

Not applicable.
